# Optical switching of radical pair conformation enhances magnetic sensitivity

**DOI:** 10.1016/j.cplett.2013.04.010

**Published:** 2013-05-30

**Authors:** Gian Giacomo Guerreschi, Markus Tiersch, Ulrich E. Steiner, Hans J. Briegel

**Affiliations:** a Institut für Quantenoptik und Quanteninformation der Österreichischen Akademie der Wissenschaften, Innsbruck, Austria; b Institut für Theoretische Physik, Universität Innsbruck, Technikerstraße 25, A-6020 Innsbruck, Austria; c Fachbereich Chemie, Universität Konstanz, D-78457 Konstanz, Germany

## Abstract

The yield of radical pair reactions is influenced by magnetic fields well beyond the levels expected from energy considerations. This dependence can be traced back to the microscopic dynamics of electron spins and constitutes the basis of chemical compasses. Here we propose a new experimental approach based on molecular photoswitches to achieve additional control on the chemical reaction and allow short-time resolution of the spin dynamics. Our proposal enables experiments to test some of the standard assumptions of the radical pair model and improves the sensitivity of a paradigmatic model of chemical magnetometer by up to two orders of magnitude.

## Highlights

• We propose to switch the conformation of radical pairs to control their reaction kinetics.
• The optical control does not interfere directly with the electron or nuclear spin dynamics.
• Our scheme may highly improve the sensitivity of chemical magnetometers.

## Graphical abstract

**Figure:**
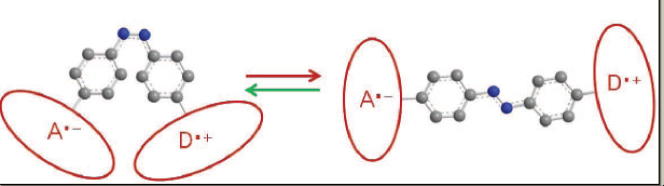

## 1 Introduction

Radical pair (RP) reactions have recently raised attention as one of the best characterized instances in the field of quantum biology [1]. Due to the strong dependence of the products’ chemical yield on the external magnetic field, they constitute one of the two main hypotheses to explain how certain species of birds use the Earth’s magnetic field to orient during migration. Recent advancements include the identification of a suitable protein, which is present in the retina and possesses the properties required by the avian magnetoreception [2,3], and the experimental demonstration that a RP reaction can be sensitive to magnetic fields as weak as the Earth’s one [4]. The formulation of an additional quantum physics perspective boosted the interest towards the study of entanglement, its decoherence and the implementation of quantum control techniques [5–8]. From a more technological perspective, these aspects are also relevant to the implementation of information processing in molecular spintronics [9,10], i.e. in electron spin systems. In both contexts, it is necessary to investigate the established RP model [11,12] on the short timescale of the electron spin dynamics and to extend the experimental studies from an ensemble approach towards the single-molecule level.

Here, we propose a new experimental setup which allows sub-nanosecond time resolution and is suitable to reduce the stochastic nature of ensemble experiments by effectively engineering the reaction kinetics. We suggest to connect the radicals via a molecular switch presenting two isomeric forms and capable of undergoing conformational changes upon the absorption of a photon. In this way, one can optically manipulate the distance between the radicals and effectively determine the instant of their re-encounter. Since the chemical reaction takes place at the moment of re-encounter, we achieve control on the reaction kinetics.

First, we present the experimental setup and discuss its feasibility with state-of-the-art technology. The consistency of timescales and the efficiency of the procedure are analyzed for the case of azobenzene as the photo-controlled bridge. Second, we show how the additional control can be exploited to improve the performance of sensors based on the RP mechanism, and in particular we apply our scheme to a specific chemical magnetometer to boost its sensitivity by two orders of magnitude.

## 2 Radical pair chemical reactions

From the broad class of chemical reactions involving intermediate radicals [13], we consider those reactions that take place in solution and involve two different chemical compounds (named A and D for acceptor and donor, respectively) free to diffuse. Typically, one of the molecules is photoexcited and, if A and D are close enough, a fast electron transfer establishes the radical pair $D^{•+}\text{"–"}A^{•-}$. The electron spins are assumed to be in the singlet state $|S\rangle =\frac{1}{\sqrt{2}}(|↑↓\rangle -|↓↑\rangle )$, while, at room temperature, the nuclear spins are in the completely mixed state. The radicals diffuse away from each other to such distances that the electron–electron interactions become negligible and the electron spin dynamics is determined only by the external magnetic field and the interaction of each electron with the surrounding nuclear spins on the respective radical [13]:

(1) $$H=\underset{m=A\text{,}D}{\sum }H_{m}=-\gamma_{e}\overset{\to }{B}\cdot \underset{m}{\sum }{\overset{\to }{S}}_{m}+|\gamma_{e}|\underset{m\text{,}j}{\sum }{\overset{\to }{S}}_{m}\cdot {\overset{ˆ}{\alpha }}_{m_{j}}\cdot {\overset{\to }{I}}_{m_{j}}$$

where $\gamma_{e}=-g_{e}\mu_{B}$ is the electron gyromagnetic ratio, ${\overset{\to }{S}}_{m}\text{,}\;{\overset{\to }{I}}_{m_{j}}$ are the electron and nuclear spin operators respectively, $\overset{\to }{B}$ is the external magnetic field, ${\overset{ˆ}{\alpha }}_{m_{j}}$ denote the hyperfine coupling tensors, and we fix ℏ=1. In case of isotropic interactions, the tensors ${\overset{ˆ}{\alpha }}_{m_{j}}$ reduce to scalars. Geminate re-encounter of the two radicals happens after a random walk in solution, and backward electron transfer completes the chemical reaction for radicals that are in a singlet state. Triplet radicals recombine to distinct triplet products.

The number of radical pairs which recombine through the singlet reaction channel is directly proportional to the time integral of the fraction of radicals in a singlet state $f_{s}(t)$ weighted by the probability of (diffusive) re-encounter: $\Phi_{s}=\int_{0}^{\infty }p_{\mathrm{re}}(t)f_{s}(t)dt$, with $p_{\mathrm{re}}(t)$ the re-encounter probability and $f_{s}(t)=\langle S|\rho_{\mathrm{el}}(t)|S\rangle$ the overlap between the singlet state and the electron spin state $\rho_{\mathrm{el}}(t)$ at time *t*. Thus, we are facing a system in which two distinct, but concurrent dynamics are taking place: The quantum evolution of the electron spin state, and the classical diffusion of the molecules $D^{+}$ and $A^{-}$. The latter one stochastically determines the duration of the former. In the literature, the probability distribution for the re-encounter time has phenomenologically been described as a single exponential $p_{\mathrm{re}}(t)={\mathrm{ke}}^{-\mathrm{kt}}$ with re-encounter rate *k* [13,14]. It is $p_{\mathrm{re}}(t)$ that we aim to control in order to modify $\Phi_{s}$ and all the related quantities.

## 3 Re-encounter probability

A large rate *k* ∼ 1 ns^−1^ indicates that most of the geminate re-encounters happen in the first few nanoseconds after the initial electron transfer, and so it becomes hard to study the electron spin dynamics at later times or to observe the dependence of the entanglement lifetime on *B* as predicted in Ref. [5]. Furthermore, a weak magnetic field effectively has no time to produce any appreciable effect and this limits the sensitivity of chemical magnetometers or compasses. In the context of avian magnetoreception, the RP is actually required [2,15] to have a small re-encounter rate *k* ∼ 1 μs^−1^. For chemical reactions involving bi-ionic radicals in solution, however, the Coulomb attraction leads to a faster re-encounter. Thus several experimental efforts have been undertaken to modify the distribution of the RP re-encounter time, mainly by imposing constraints on the geometry of the system by linking the radicals with a flexible chain [16,17] or restricting the available diffusion volume using micelles [18]. Although these experimental studies confirm a substantial modification of $p_{\mathrm{re}}(t)$ in these systems, they are still interpreted within the phenomenological exponential model with rate *k* ∼ 0.1–1 μs^−1^ essentially determined by properties (like solvent viscosity or chain length) that cannot be changed continuously or even independently, and whose effect on the rate *k* is not easy to predict quantitatively. To overcome these difficulties, we propose to use optical switches, in this way combining geometrical constraints with the possibility of optically controlling them.

## 4 Photo-controlled radicals

**Figure 1:**
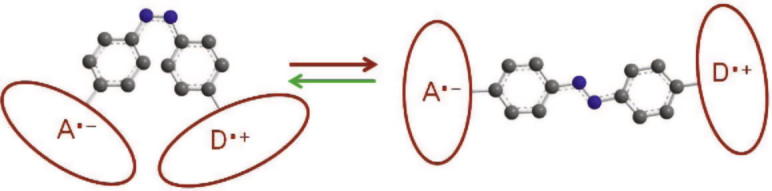
Control of the re-encounter probability by optically switching between conformations of a bridge molecule (here azobenzene).

The distance of the radicals strongly affects both the possibility of charge recombination and the electron spin dynamics. At small separations, the electron transfer is favored by the proximity of the radicals, while the direct electron–electron coupling inhibits the spin dynamics. In fact, the exchange interaction determines an energy gap between the singlet state and the triplet states which suppresses the spin interconversion. Since such interaction decreases exponentially with increasing distance between the radicals, for the moderate separation reached in bi-ionic reaction in solution they are typically neglected (as expressed in Eq. 1). Consistently, at large distances the dynamics is determined by the Zeeman and hyperfine interaction only, whereas the electron transfer is practically forbidden. Here, we propose to optically control the inter-radical separation by chemically attaching A and D to the two endings of a molecule exhibiting two isomeric forms and an isomerization pathway which is photo-activated. Such molecules possess the property of reversibly switching between a straight and a contracted structure upon the absorption of a photon of suitable wavelength. The two isomeric forms effectively impose a ‘closed’, respectively ‘open’, configuration for the radicals, see Figure 1, that we are able to change in a reversible way by means of short laser pulses. Examples of photoswitchable compounds are fulgides, diarylethenes or azobenzene derivatives [19].

Via the optical manipulation of the opening and closing of the linking bridge, it is in principle possible to directly control the instant of RP creation, the exact duration of the quantum dynamics for the electron spins, and the instant of the final recombination. The implementation of such a scheme for an ensemble of molecules is ideally as follows: initially all the bridges are closed and the radicals not yet formed, then the RP are created by excitation via a laser pulse (standard technique applied for example in Ref. [4,20]), and immediately after that, a second laser pulse triggers the isomerization and separates the radicals. The electron spin state can then evolve according to the Hamiltonian (1) until a third laser pulse will close the bridges and lead to the final recombination. In this scheme, every molecule undergoes a single cycle of isomerization: From the closed form to the open one, and back to the closed form again. It is desirable, but not strictly necessary, that all the three processes (i.e. the radical pair creation and the two opposite isomerization processes) could be selectively triggered by laser pulses of different wavelengths.

For pulse durations much shorter than the timescale of the electron spin evolution, the re-encounter probability can be described by a peaked distribution $p_{\mathrm{re}}(t)\propto \delta (t-\tau_{\mathrm{re}})$, with $\tau_{\mathrm{re}}$ being the time elapsed between the second and third pulse and ‘∝’ indicating the proportionality with respect to the actual photoisomerization yield. Such peaked distribution allows to monitor the electron spin dynamics at the precise instant $\tau_{\mathrm{re}}$, which can be chosen in a wide interval ranging from a few nanoseconds to several tens of microseconds (for later times one has to take into account spin–lattice relaxation and other decoherence sources). The proposed scheme can be straightforwardly adapted to engineer more complicated and structured re-encounter probability distributions: a series of pulses creates a re-encounter probability exhibiting separated peaks, while continuous illumination gives rise to an exponential distribution (here not phenomenologically assumed, but externally imposed) whose rate is directly proportional to the light intensity. Combining laser pulses, dark intervals, and continuous illumination with adjustable light intensity, the ability of engineering a specific $p_{\mathrm{re}}(t)$ is practically limited by technical restrictions (e.g. the laser intensity or the speed of light modulation) and by the absorption and isomerization properties of the photoswitches (e.g. the selectivity with which an ‘opening pulse’ does not cause spurious ‘closing events’, and vice versa). Realistically, a fraction of the photoswitches may not undergo photoisomerization, but, for thermally stable isomers, this means that the corresponding radicals simply do not contribute to the chemical reaction.

An alternative scenario is given by photoswitches that do not change their conformation, but rather change their conductance, passing from an insulating to a conductive state and vice versa [21,22]. In the case of backward electron transfer through the linker, the isomerization of the linker corresponds to switching on/off the coupling between the two radicals at its ends.

## 5 Case study: azobenzene

To explore the actual feasibility of our proposal, we consider the concrete example of azobenzene as the photoswitching molecular bridge (for more details see supplementary material). Azobenzene is an organic molecule that occurs in two isomeric forms, trans and cis azobenzene, having a stretched, respectively contracted, structure. The synthesis of azobenzene derivatives having different chemical functional groups is routinely performed [23–25]. Preliminary checks will have to verify that the chemistry of the RP is not altered by the presence of the azobenzene and that the constraints on the RP distance are satisfied.

The processes involved in our proposal need to satisfy a hierarchy of timescales: the electron spin dynamics determines the reference timescale to be ∼1–10 ns (for typical values of the external magnetic field and hyperfine couplings, i.e. ∼0.1–1 mT) with respect to which the spin relaxation (either dissipation or dephasing) and the thermally driven isomerization have to be slow, while the radical creation and photoisomerization mechanism have to be fast. Typically, spin–lattice relaxation takes ∼1–10 μs at room temperature, while photo-excitation of the donor and successive electron transfer happens in a few picoseconds. Radicals were generated by a 7 ns pulse in [4], but even RP creation in less than one picosecond has been achieved in experiments with femtosecond laser pulses [26]. In the case of azobenzene, the isomers can be considered completely stable at room temperature [27] whereas the photoisomerization takes only a few picoseconds [28,29]. The timescale over which we can modulate the re-encounter probability depends on the strength and duration of the optical pulses: Estimates in Ref. [30] suggest that a single pulse of a duration of ≃10 ps achieves photoisomerization yields of 30–40%.

## 6 Application: chemical magnetometry

The control of the RP re-encounter probability finds a direct application to improve the performance of chemical devices. Here, we show how a simple-to-implement control scheme highly enhances the sensitivity of a model chemical magnetometer by up to two orders of magnitude. The basic idea behind a chemical magnetometer is that, since a change in the magnetic field modifies the amount of singlet products, one can reverse the reasoning and measure the chemical yield to estimate *B*. Intuitively, the magnetic sensitivity is high when a small change in the magnetic field intensity produces large effects on the singlet yield. Formally, it is defined as:

(2) $$\Lambda_{s}(B)\equiv \frac{\partial \Phi_{s}(B)}{\partial B}=\int_{0}^{\infty }p_{\mathrm{re}}(t)g_{s}(B\text{,}t)dt\text{,}$$

with $g_{s}(B\text{,}t)\equiv \frac{\partial f_{s}(B\text{,}t)}{\partial B}$ being the instantaneous magnetic sensitivity. The functional form of $f_{s}(B\text{,}t)=\langle S|\rho_{\mathrm{el}}(t)|S\rangle$ strongly depends on the specific realization of the radical pair, in particular on the number of the surrounding nuclear spins. Here, we consider a radical pair in which the first electron spin is devoid of hyperfine interactions, while the second electron spin interacts isotropically with one spin-1 nucleus, e.g. nitrogen. In the context of the chemical compass (i.e. when the task is determining the magnetic field direction through anisotropic hyperfine interactions), an analogous configuration (with only one spin-1/2 nucleus) has been proposed [3], and numerically characterized [8], as being optimal: Additional nuclear spins would perturb the intuitive ‘reference and probe’ picture. The Hamiltonian then simplifies to $H=-\gamma_{e}B(S_{1}^{\left(z\right)}+S_{2}^{\left(z\right)})+|\gamma_{e}|\alpha {\overset{\to }{S}}_{2}\cdot \overset{\to }{I}$, where α is the isotropic hyperfine coupling.

**Figure 2:**
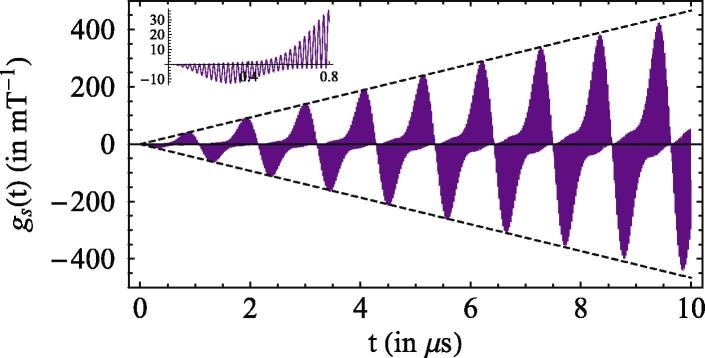
Instantaneous magnetic sensitivity for *α* = 1 mT and $B=\frac{\alpha }{20}$. The peaks of the envelope grow linearly with time and the envelope’s shape is approximately periodic with period $\tau_{\text{envel}}=\frac{3\pi }{|\gamma_{e}|B}\approx 1.07\;\mu \text{s}$. Inside the envelope, $g_{s}(t)$ oscillates with period $≃\frac{4\pi }{3|\gamma_{e}|\alpha }\approx 23.8\;\text{ns}$ (see inset).

We evaluate the sensitivity in the regime of long spin–lattice relaxation times and weak magnetic fields, i.e. for B≪α, noting that, in practical applications in which the *B* field varies around a large ‘off-set’ value, one can reproduce such conditions by applying a suitable external field for compensation. For the realistic choice α≃1.0mT, the Earth magnetic field is indeed weak: $B_{\text{Earth}}≃\alpha /20$. We have obtained an analytical expression for the instantaneous magnetic sensitivity $g_{s}(B\text{,}t)$ as given in the supplementary material, whose behavior, in the regime B≪α, is shown in Figure 2.

**Figure 3:**
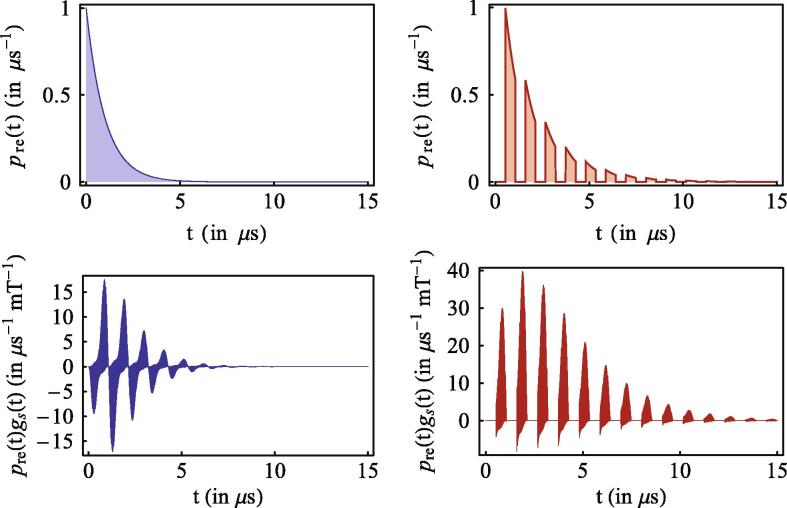
Re-encounter probability (top) and integrand for the magnetic sensitivity (bottom) over time. The blue curve (left) corresponds to the phenomenological exponential model, the red curve (right) to the optically controlled case. Note that we have chosen the laser intensity such that *k*_prot_ = *k* = 1 μs^−1^ to make the comparison more direct. In general, while *k* is determined by the radicals and properties of the solution, $k_{\text{prot}}$ is a tunable parameter of the protocol. (For interpretation of the references to color in this figure legend, the reader is referred to the web version of this article.)

When integrated on timescales longer than the effective ‘period’ of the envelope, $\tau_{\text{envel}}=\frac{3\pi }{|\gamma_{e}|B}$, the positive and negative contributions cancel each other and drastically reduce the ultimate sensitivity $\Lambda_{s}(B)$. A solution is to engineer the re-encounter probability in such a way that the radical pairs are allowed to recombine only when $g_{s}(B\text{,}t)⩾0$. Such desirable $p_{\mathrm{re}}(t)$ is obtained applying weak light pulses (for the final step of ‘closing’ the photoswitchable bridge) interspersed with equally long dark intervals, both of duration $\tau ≃\tau_{\text{envel}}/2$. Considering the realistic parameters of Figure 2, one obtains τ≈0.54μs corresponding to a laser repetition of around 1 MHz. The corresponding re-encounter probability is a piecewise exponential function with decay rate $k_{\text{prot}}$ directly proportional to the light intensity. The phenomenological and controlled re-encounter probabilities are shown in Figure 3 together with the corresponding integrand involved in the evaluation of the magnetic sensitivity (2).

**Figure 4:**
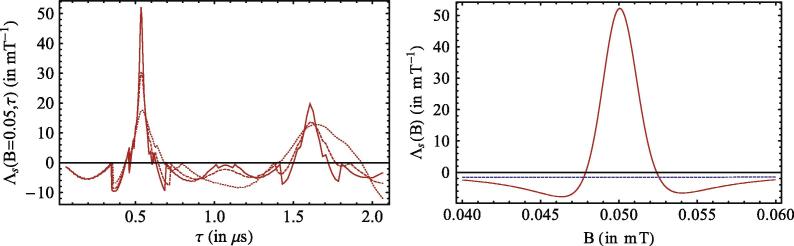
Left: Magnetic sensitivity $\Lambda_{s}$*vs.* pulse duration τ. *B* = 0.05 mT and 1/*k*_prot_ = 2 μs (solid), 1 μs (dashed), 0.5 μs (dotted). Right: Magnetic sensitivity $\Lambda_{s}$*vs.* field intensity *B* for the exponential model with 1/*k* = 10 ns ($\Lambda_{s}\approx -0.03\;{\text{mT}}^{-1}$, solid, blue), 1/k=100ns ($\Lambda_{s}\approx -1.59\;{\text{mT}}^{-1}$, dashed, blue), 1/*k* = 2 μs ($\Lambda_{s}\approx -0.05\;{\text{mT}}^{-1}$, dotted, blue), and for our proposal with pulse duration $\tau^{\prime }=0.535\;\mu \text{s}$ (solid, red). Parameters as in Figure 2. (For interpretation of the references to color in this figure legend, the reader is referred to the web version of this article.)

The experimental protocol that we suggest is characterized by the sole parameter τ, which represents both the pulse duration and the waiting time between two consecutive pulses. In Figure 4 (left) we plot the (integrated) magnetic sensitivity as a function of τ, and observe the presence of a sharp peak located in correspondence to the resonance condition $\tau^{\prime }≃\tau_{\text{envel}}/2$. As expected, a weaker resonance gives rise to a second peak at $\tau ≃3\tau^{\prime }$. The choice of a suitable τ is then essential to obtain an enhancement of the magnetic sensitivity: Since the appropriate $\tau^{\prime }$ is inversely proportional to the intensity of the external field, a rough estimate of it is required. If an approximated value $B_{\text{app}}≃B$ of the magnetic field is known, one can run the protocol with the corresponding value of $\tau^{\prime }≃\frac{3\pi }{2|\gamma_{e}|B_{\text{app}}}$. Otherwise, if the value of the external magnetic field is completely unknown, we suggest a series of experimental runs which scans over a suitable range of pulse durations. From an irregular landscape, a sharp peak centered around a certain $\tau^{\prime }$ will emerge, providing a first estimate of the field intensity: Consistently with the expressions given above, one obtains $B_{\text{estim}}=\frac{3\pi }{2|\gamma_{e}|\tau^{\prime }}$. In both cases, the precise value of *B* is determined by applying pulses of duration $\tau^{\prime }$. Figure 4 (right) predicts that the optical control of $p_{\mathrm{re}}(t)$ enhances the magnetic sensitivity up to two orders of magnitude with respect to the phenomenological exponential model over a broad range of rate *k*. We observe that similar enhancements are obtained for several $k_{\text{prot}}$ satisfying $k_{\text{prot}}⩽\tau^{-1}$, and for a single nuclear spin-1/2 together with a suitably modified protocol (see supplementary material).

A few remarks are necessary. In principle, to associate a unique magnetic field *B* to the experimentally observed singlet yield, the function $\Phi_{s}(B)$ must be invertible or, equivalently in this case, strictly monotonic. Any specific realization of a chemical magnetometer presents several functional windows associated with a range of magnetic field intensities in which the derivative $\frac{\partial \Phi_{s}(B)}{\partial B}$ does not change sign. Inside any functional window, the uncertainty in the estimation of *B* is inversely proportional to the corresponding magnetic sensitivity $\Lambda_{s}(B)$. Since the singlet yield $\Phi_{s}(B)$ takes only values in [0,1], the area below the sensitivity curve must sum up to $|\Delta \Phi_{s}|⩽1$ when integrated on any functional window. Therefore, in the best case, the range of field intensities that lead to high sensitivity (and in which, we remind the reader, the sensitivity should not change its sign) has a width that is inversely proportional to the average value of sensitivity achieved. For realistic functional forms of $\Lambda_{s}(B)$, it is also inversely proportional to the maximum value. For a chemical magnetometer, a high sensitivity requires a correspondingly ‘narrow’ functional range. The good news is that, with our protocol, we can move such window and center it on the actual magnetic field.

Finally, let us estimate how the sensitivity in Figure 4 (right) compares to the best performance achievable by a chemical magnetometer, possibly in connection with an arbitrarily complicated pulse scheme: For a functional range between 0.048 mT and 0.052 mT, i.e. of width 0.004 mT, as taken from the figure, the average sensitivity cannot be greater than 250 mT^−1^. The average sensitivity in the given range amounts to about 25 mT^−1^ which means that our magnetometer is optimal within a factor of 10. This is remarkable since the proposed pulse sequence is characterized by the sole parameter τ and because such time is much longer than the timescale of the electron spin dynamics. As a matter of fact, it is comparable to $\frac{1}{\left|\gamma_{e}B\right|}$ which is the longest timescale involved.

## 7 Conclusions

We have proposed a new experimental approach which enables the control of the re-encounter probability of radical pairs in solution via light pulses. This allows one to study the electron spin dynamics on the short timescale of its evolution and enlarges the information accessible in spin chemistry experiments. Reducing the stochastic nature of ensemble and time averages will contribute to a better understanding of the radical pair mechanism itself.

So far, two types of quantum control schemes have been explored in chemistry. (i) In addition to static magnetic fields, the spin chemistry of radical pairs has been controlled by resonant microwave fields (reaction yield detected magnetic resonance, RYDMR) [31,32,20,33]. This type of control coherently interacts with the spin degrees of freedom of radical pairs. Recent theoretical work has added other variants to this type of control with direct manipulation of the electron spins [5,7,8]. (ii) Photoreactions, in particular photodissociation reactions of small molecules, have been subject to coherent laser control with shaped fs-pulses [34,35]. In this situation, it is the quantum dynamics of valence electrons and nuclear motion coupled to them that is directly acted on. The present scheme represents a novel type of quantum control in chemistry. It does not interfere directly with the electronic or spin degrees of freedom of a radical pair, but it switches parts of its Hamiltonian (exchange interaction) and the reaction constant. In our model, the control is achieved through the photo-chemical effect of light on the conformation of the radical pair, i.e. in principle by a purely ‘mechanical’ effect that could be also obtained by some other kind of micro-mechanics, perhaps in an STM tip. Recently, Wasielewski and co-workers [36] have experimentally demonstrated similar effects using a completely different approach, in which the distance between the two electrons was changed by triggering a secondary electron transfer.

Regarding technological applications, the proposed experimental scheme offers an approach to increase the sensitivity of man-made magnetic-field sensors based on chemical reactions. This we have demonstrated with a suitable radical pair connected by a photoswitchable bridge. We have presented a simple protocol, which enhances the sensitivity of the chemical magnetometer by up to two orders of magnitude. Remarkably, this scheme can be implemented using weak laser pulses, which have a duration much longer than the timescale given by the electron spin dynamics and, indeed, comparable to the longest one as defined by the weak magnetic field $\frac{1}{|\gamma_{e}|B}$.
