# Supplementary material for: Optical switching of radical pair conformation enhances magnetic sensitivity
Source: Chem Phys Lett. 2013 May 30;572:106–10. doi: 10.1016/j.cplett.2013.04.010 (PMC4375724; doi:10.1016/j.cplett.2013.04.010)
Supplement: Supplementary file 1 [file mmc1.pdf]

Supplementary material

Optical control of radical pair reactions enhances magnetic sensitivity

---

Gian Giacomo Guerreschi, Markus Tiersch, Ulrich E. Steiner, and Hans J. Briegel

Case study: azobenzene

Azobenzene is an organic molecule composed of two phenyl rings linked by a N=N double bond, and occurs in two isomeric forms, trans and cis azobenzene, having a stretched, respectively contracted, structure. The synthesis of azobenzene derivatives having different chemical functional groups extending from the phenyl rings is routinely performed [1, 2]. Preliminary checks have to verify that the chemistry of the radical pair is not drastically altered by the presence of the azobenzene and that the constraints on the RP distance are satisfied.

From the Letter:
For a realistic implementation of our proposal, the involved processes need to satisfy a hierarchy of time scales: The electron spin dynamics determines the reference time scale to be $\sim 1-10\,ns$ *(for typical values of the external magnetic field and hyperfine couplings, i.e.* $\sim 0.1-1\,mT$*) with respect to which the spin lattice relaxation and the thermally driven isomerization have to be slow, while the radical creation and photoisomerization mechanism have to be fast.*

In the case of azobenzene, the isomers can be considered completely stable at room temperature on the time scale of seconds [3], while experimental studies with azobenzene in solution afforded S_1 excited state lifetimes of trans-azobenzene of $\sim 2.5\,$ps when excited without excess vibrational energy [4] and $\sim 0.5\,$ps when from higher vibrational states of S_1 populated after internal conversion from S_2 [5]. Consistently, *ab-initio* numerical studies estimated the time required by the photoisomerization mechanism to be $\leq 1\,$ps in the gas phase [6, 7]. Concerning the duration of the optical pulses to induce isomerization: The estimates in Ref. [8] suggest that a single pulse of 150 fs FWHM at 439 nm and having an energy of 200 nJ leads to 3% cis-to-trans photoisomerization yield. Accordingly, a single pulse of duration $\simeq 10$ ps and analogous intensity can achieve photoisomerization yield of $30-40\%$. For comparison with the process of RP creation, in [9] radicals have been generated by a 7 ns , 5 mJ pulse.

Singlet fidelity and instantaneous magnetic sensitivity

As a model chemical magnetometer, in the Letter we consider a radical pair involving a single nuclear spin with isotropic hyperfine interaction. In this case the Hamiltonian simplifies to:

$$H = -\gamma_e B(S_1^{(z)} + S_2^{(z)}) + |\gamma_e|\alpha \vec{S}_2 \cdot \vec{I},$$

where $\gamma_e = -g_e\mu_B$ is the electron gyromagnetic ratio, $\vec{S}_m$, $\vec{I}$ are the electron and nuclear spin operators respectively, B is the external magnetic field here chosen along the z direction, α is the isotropic hyperfine coupling, and we fix $\hbar = 1$.

$f_s(t) = \langle S|\, \rho_{el}(t)\, |S\rangle$ represents the overlap between the singlet state and the electron spin state $\rho_{el}(t)$ at time t, while its derivative with respect to the magnetic field intensity gives

the instantaneous magnetic sensitivity $g_s(B,t) \equiv \frac{\partial f_s(B,t)}{\partial B}$. The ultimate sensitivity is obtained integrating $g_s(B,t)$ weighted by the re-encounter probability $p_{re}(t)$:

$$\Lambda_s(B) \equiv \frac{\partial \Phi_s(B)}{\partial B} = \int_0^\infty p_{re}(t) g_s(B,t) \mathrm{d}t,$$

In the case of a single nuclear spin 1/2 (as *e.g.* for a hydrogen atom), the explicit expressions of the singlet fidelity is:

$$f_s\left(\frac{t}{|\gamma_e|}\right) = \frac{1}{8}\left\{\left(4 - \frac{\alpha^2}{A^2}\right) + \frac{\alpha^2}{A^2}\cos(At) + \frac{A+B}{A}\left[\cos\left(\frac{\alpha+B-A}{2}t\right) + \cos\left(\frac{\alpha-B+A}{2}t\right)\right] + \right.$$
$$\left. + \frac{A-B}{A}\left[\cos\left(\frac{\alpha+B+A}{2}t\right) + \cos\left(\frac{\alpha-B-A}{2}t\right)\right]\right\}$$

with $A \equiv \sqrt{\alpha^2 + B^2}$. In the limit $b \equiv \frac{B}{\alpha} \ll 1$, the above expression and the instantaneous magnetic sensitivity simplify to:

$$f_s\left(\frac{t}{|\gamma_e|}\right) \approx \frac{3}{8} + \frac{1}{8}\cos\left(\alpha\left(1+\frac{b^2}{2}\right)t\right) + \frac{1}{4}\cos\left(\frac{Bt}{2}\right)\left[\cos\left(\alpha\left(1+\frac{b^2}{4}\right)t\right) + \cos\left(\frac{B}{4}bt\right)\right] +$$
$$+ b\,\frac{1}{4}\sin\left(\frac{Bt}{2}\right)\left[\sin\left(\alpha\left(1+\frac{b^2}{4}\right)t\right) + \sin\left(\frac{B}{4}bt\right)\right] + \ldots$$

$$g_s\left(\frac{t}{|\gamma_e|}\right) \approx -\frac{1}{8}\left\{\ldots + t\left[2\left(1-b^2\right)\cos\left(\frac{\alpha t}{2}\right)\sin\left(\frac{Bt}{2}\right)\cos\left(\frac{\alpha}{2}\left(1+\frac{b^2}{2}\right)t\right) + b\,\sin\left(\alpha\left(1+\frac{b^2}{2}\right)t\right)\right] + \ldots\right\}$$

where $\approx$ means that we keep amplitudes up to $\mathcal{O}(b)$ and frequency up to $\mathcal{O}(b^2)$. For $g_s(B,t)$ we keep only the terms proportional to t, but up to $\mathcal{O}(b^2)$ also in the amplitudes.

In the case of a single nuclear spin 1 (as *e.g.* for a nitrogen atom), the explicit expressions are more complicated and not particularly significant. The singlet fidelity, for a given B field, is:

$$f_s\left(\frac{t}{|\gamma_e|}\right) = \frac{1}{2} + \frac{\alpha^4}{6\eta_+^2\eta_-^2}\left\{\frac{\eta_+^2\eta_-^2}{\alpha^4}\left[\cos\left(\frac{2B+3\alpha}{4}t\right)\cos\left(\frac{\eta_-}{\sqrt{2}}t\right) + \cos\left(\frac{2B-3\alpha}{4}t\right)\cos\left(\frac{\eta_+}{\sqrt{2}}t\right) + \cos(Bt)\cos\left(\frac{\eta_-}{\sqrt{2}}t\right)\cos\left(\frac{\eta_+}{\sqrt{2}}t\right)\right] + \right.$$
$$- \frac{\eta_+^2}{\alpha^2}\sin^2\left(\frac{\eta_-}{\sqrt{2}}t\right) - \frac{\eta_-^2}{\alpha^2}\sin^2\left(\frac{\eta_+}{\sqrt{2}}t\right)$$
$$- \frac{\eta_+\eta_- B_+ B_-}{\alpha^4}\cos(Bt)\sin\left(\frac{\eta_-}{\sqrt{2}}t\right)\sin\left(\frac{\eta_+}{\sqrt{2}}t\right)$$
$$+ \frac{\eta_+^2\eta_- B_-}{\alpha^4}\left[\sin(Bt)\sin\left(\frac{\eta_-}{\sqrt{2}}t\right)\cos\left(\frac{\eta_+}{\sqrt{2}}t\right) + \sin\left(\frac{2B+3\alpha}{4}t\right)\sin\left(\frac{\eta_-}{\sqrt{2}}t\right)\right] +$$
$$\left. + \frac{\eta_+\eta_-^2 B_+}{\alpha^4}\left[\sin(Bt)\cos\left(\frac{\eta_-}{\sqrt{2}}t\right)\sin\left(\frac{\eta_+}{\sqrt{2}}t\right) + \sin\left(\frac{2B-3\alpha}{4}t\right)\sin\left(\frac{\eta_+}{\sqrt{2}}t\right)\right]\right\}$$

where $B_\pm \equiv \frac{2B\pm\alpha}{2\sqrt{2}}$ and $\eta_\pm \equiv \sqrt{\alpha^2 + B_\pm^2}$.

Enhanced magnetic sensitivity in the presence of a spin-1/2 nucleus

In the paragraph "*Application: chemical magnetometry*" of the Letter, we propose a protocol to enhance the sensitivity of a chemical magnetometer by engineering the re-encounter probability of the two radicals involved. To do so, we consider the specific case of a radical pair in which one electron spin is avoid of hyperfine interactions and the second only interacts with a spin-1 nucleus.

Here, we show that similar results can indeed be obtained also for a single nuclear spin-1/2. In this case, the instantaneous magnetic sensitivity for an isotropic hyperfine coupling $\alpha = 1.0\,\mathrm{mT}$ and magnetic field $B = 0.05\,\mathrm{mT}$ (as week as the Earth's one) is shown in Fig. 1.

The protocol is now slightly different since, in a periodic way over long time scales, $g_s(B,t)$ quickly oscillates between positive and negative contributions. For this reason, the alternating

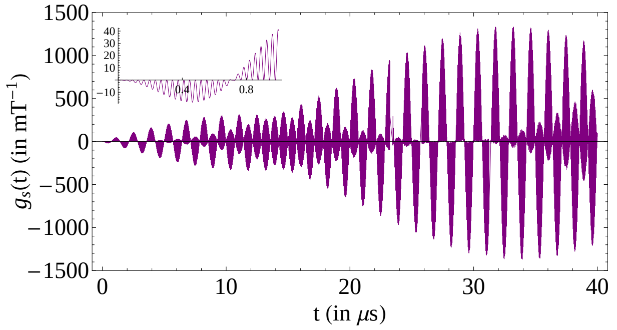

Figure 1: Instantaneous magnetic sensitivity for $\alpha = 1$ mT and $B = \frac{\alpha}{20}$. The fast frequency of oscillation is shown in the inset.

laser scheme (with pulses of duration τ interspersed with dark, equally long, waiting times) has to be modified introducing longer pauses to avoid re-encounter events whenever the sign of $g_s(B,t)$ changes too fast (see Fig. 2).

Finally, Fig. 3 summarizes the results in a way analogous to Fig. 4 of the Letter, with the difference that the peak of sensitivity is now determined by the resonance condition $\tau' \simeq \frac{2\pi}{|\gamma_e|B}$. Notice that the single peak is now divided into three narrower peaks, see Fig. 3 (right): The fact that all the three peaks correspond to positive values of sensitivity guarantees that the singlet yield increases monotonically for increasing magnetic field in the range $B \in [0.048\,\mathrm{mT}, 0.052\,\mathrm{mT}]$.

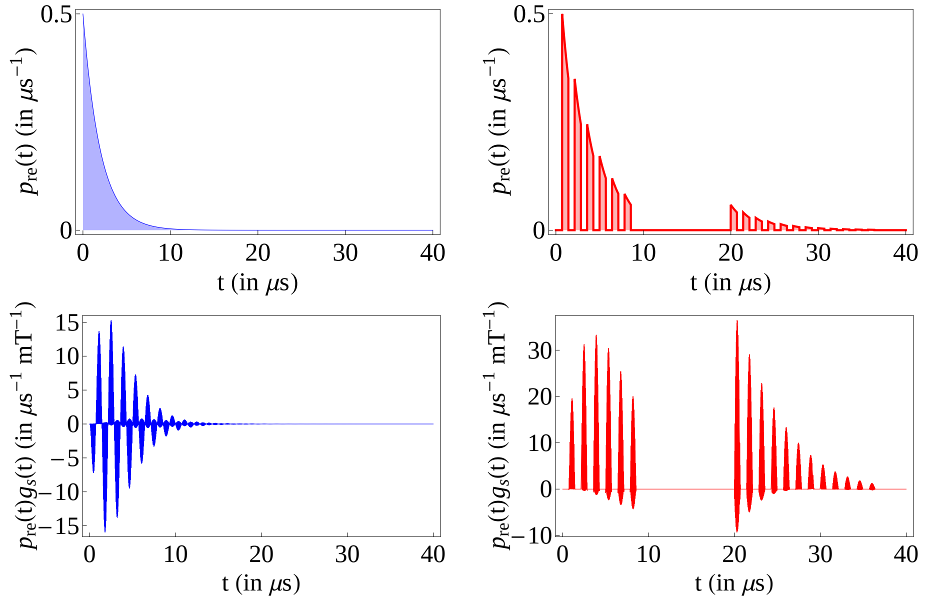

Figure 2: Re-encounter probability (top) and integrand for the magnetic sensitivity (bottom) over time. The blue curve (left) corresponds to the phenomenological exponential model, the red curve (right) to the optically controlled case. As in the Letter, we have chosen the laser intensity such that $k_{prot} = k = 2\mu\mathrm{s}^{-1}$ to make the comparison more direct. In general, while k is determined by the radicals and properties of the solution, k_{prot} is a tunable parameter of the protocol.

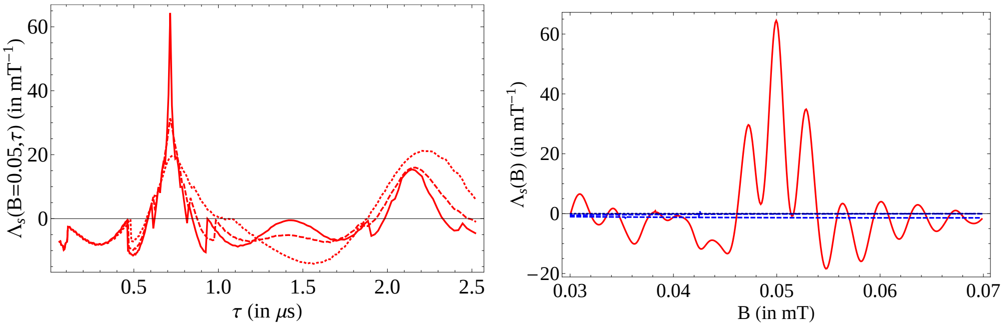

Figure 3: Left: Magnetic sensitivity Λ_s *vs.* pulse duration τ. $B = 0.05\,\mathrm{mT}$ and $1/k_{prot} = 2\,\mu\mathrm{s}$ (solid), $1\,\mu\mathrm{s}$ (dashed), $0.5\,\mu\mathrm{s}$ (dotted). Right: Magnetic sensitivity Λ_s *vs.* field intensity B for the exponential model with $1/k = 10\,\mathrm{ns}$ ($\Lambda_s \approx -0.01\,\mathrm{mT}^{-1}$, solid, blue), $1/k = 100\,\mathrm{ns}$ ($\Lambda_s \approx -1.34\,\mathrm{mT}^{-1}$, dashed, blue), $1/k = 2\,\mu\mathrm{s}$ ($\Lambda_s \approx -0.12\,\mathrm{mT}^{-1}$, dotted, blue), and for our proposal with pulse duration $\tau' = 0.715\,\mu\mathrm{s}$ (solid, red). Parameters as in Fig. 1.

References

[1] T. Hugel *et al.*, Science **296**, 1103 (2002).

[2] C. C. Hofmann *et al.*, J. Chem. Phys. **131**, 144512 (2009).

[3] H. Rau and E. Lüddecke, J. Am. Chem. Soc. **104**, 1616 (1982).

[4] I. K. Lednev *et al.*, Chem. Phys. Lett. **290**, 68 (1998).

[5] T. Fujino, S. Yu. Arzhantsev, and T. Tahara J. Phys. Chem. A **105**, 8123 (2001).

[6] M. Böckmann, N. L. Doltsinis, and D. Marx, Phys. Rev. E **78**, 036101 (2008).

[7] M. Böckmann, N. L. Doltsinis, and D. Marx, Angew. Chem. Int. Ed. **49**, 3382 (2010).

[8] A. Yamaguchi *et al.*, Applied Surface Science **255**, 9864 (2009).

[9] K. Maeda *et al.*, Nature **453**, 06834 (2008).
